# Relationship between quality of life and behavioural disorders in children with persistent asthma: a Multiple Indicators Multiple Causes (MIMIC) model

**DOI:** 10.1038/s41598-020-62264-9

**Published:** 2020-04-24

**Authors:** Laura Montalbano, Giuliana Ferrante, Silvia Montella, Giovanna Cilluffo, Antonio Di Marco, Sara Bozzetto, Emanuela Di Palmo, Amelia Licari, Lucia Leonardi, Valeria Caldarelli, Michele Ghezzi, Stefania La Grutta, Franca Rusconi, the Italian Pediatric Severe Asthma Network (IPSAN) Program of Italian Paediatric Respiratory Society (IPRS), S. Amarri, S. Barni, A. Capizzi, F. Cardinale, S. Carraro, S. Cazzato, R. Cutrera, S. Di Pillo, M. Duse, G. Fenu, A. Kantar, S. Leonardi, E. Lombardi, G. L. Marseglia, L. Nosetti, E. Novembre, M. F. Patria, G. Piacentini, G. Pisi, G. Ricci, O. Sacco, F. Santamaria, L. Tenero, M. A. Tosca, M. C. Tripodi, A. Volpini

**Affiliations:** 1 National Research Council of Italy, Institute for Research and Biomedical Innovation, IRIB, Via Ugo La Malfa 153, 90146 Palermo, Italy; 2 Department of Health Promotion, Mother and Child Care, Internal Medicine and Medical Specialities, University of Palermo, Palermo, Italy; 3 Department of Translational Medical Sciences, Federico II University, Via Sergio Pansini 5, 80131 Naples, Italy; 4 Pediatric Pulmonology and Sleep & Long Term Ventilation Unit, Academic Department Pediatric Hospital “Bambino Gesù”, Piazza S. Onofrio 4, Rome, Italy, 00165 Roma, Italy; 5 Division of Emergency Medicine, Department of Women’s and Children’s Health, University of Padova, Padova, Italy; 6 Pediatric Unit, Department of Medical and Surgical Sciences, University of Bologna, 40138 Bologna, Italy; 7 Pediatric Clinic, Fondazione IRCCS Policlinico San Matteo, University of Pavia, piazzale Golgi 19, 27100 Pavia, Italy; 8 Department of Paediatrics, “Sapienza” University of Rome, Rome, Italy; 9 Pediatric Unit, Department of Obstetrics, Gynecology and Pediatrics, Azienda USL - IRCCS, Azienda USL-IRCCS, Viale Risorgimento, 80, 42123, Reggio Emilia, Italy; 10 Department of Pediatrics, Ospedale dei Bambini, University of Milan, Milan, Italy; 11 Unit of Epidemiology, ‘Anna Meyer’ Children’s University Hospital, Viale Pieraccini 24, 50139 Florence, Italy; 12 Department of Mother and Child, Azienda USL-IRCCS, Viale Risorgimento 80, 42123 Reggio Emilia, Italy; 13 Allergy Unit, Department of Pediatrics, Meyer Children’s University Hospital, 50139 Florence, Italy; 14 Department of Pediatrics, Pulmonary and Allergy Disease Pediatric Unit and Cystic Fibrosis Center, IRCCS Istituto Giannina Gaslini, Genoa, Italy; 15 Department of Pediatrics and Emergency, Pediatric Allergy and Pulmunology Unit, Azienda Ospedaliera-Universitaria “Consorziale-Policlinico”, Ospedale Pediatrico Giovanni XXIII, Bari, Italy; 16 Women’s and Children’s Health Department, University of Padova, via Giustiniani 3, 35128 Padova, Italy; 17 Pediatric Unit, Department of Mother and Child Health, Salesi Children’s Hospital, Ancona, Italy; 18 Pediatric Pulmonology and Sleep & Long Term Ventilation Unit, Academic Department Pediatric Hospital “Bambino Gesù”, Piazza S. Onofrio 4, 00165 Rome, Italy; 19 Department of Pediatrics, University of Chieti, Chieti, Italy; 20 Department of Pediatrics, Sapienza University, Rome, Italy; 21 Pediatric Pulmonary Unit, Meyer Pediatric University Hospital, Florence, Italy; 22 Centro Pediatrico dell’Asma e della Tosse, Istituti Ospedalieri Bergamaschi, Ponte San Pietro, Bergamo, Italy; 23 Pediatric Broncho-Pneumology and Cystic Fibrosis Unit, Department of Clinical and Experimental Medicine, University of Catania, Catania, Italy; 24 Pediatric Clinic, Department of Pediatrics, Fondazione IRCCS Policlinico San Matteo, University of Pavia, 27100 Pavia, Italy; 25 Division of Pediatrics, “F. Del Ponte” Hospital, University of Insubria, Varese, Italy; 26 Allergy Unit, Department of Pediatrics, Anna Meyer Children’s University Hospital, 50139 Florence, Italy; 27 Pediatric Highly Intensive Care Unit, Department of Pathophysiology and Transplantation, Università degli Studi di Milano, Fondazione IRCCS Ca’ Granda Ospedale Maggiore Policlinico, Via Commenda 9, 20122 Milan, Italy; 28 Department of Surgery, Dentistry, Paediatrics and Gynaecology, University of Verona, Verona, Italy; 29 Cystic Fibrosis Center, University Hospital Parma, Italy; 30 Pediatric Unit, Department of Medical and Surgical Sciences, University of Bologna, Via Massarenti 11, 40138 Bologna, Italy; 31 Pediatric Pneumology and Respiratory Endoscopy Gaslini Children Hospiral Via Gerolamo Gaslini 5, 16147 Genova, Italy; 32 Department of Translational Medical Sciences, Federico II University, Via Pansini 5, 80131 Naples, Italy; 33 Department of Pediatrics, Allergy Center, Istituto Giannina Gaslini (IRCCS), Genoa, Italy; 34 Cystic Fibrosis Unit, Departmet of Pediatrics, University Hospital, Parma, Italy; 35 Department of Pediatrics, Children’s Hospital “G. Salesi”, AOU Ospedali Riuniti Ancona, Ancona, Italy

**Keywords:** Psychology, Human behaviour, Medical research, Paediatric research

## Abstract

Knowledge on multiple interdependences between quality of life (QoL) and behavioural problems in relation to asthma severity and control is undetermined. The aims of the study were: (i) to assess the relationship of QoL and behavioural problems with asthma severity and control (ii) to predict children’s “abnormal/borderline” status with variation in QoL. For these purposes a multicenter case-control study on 47 Severe Asthma (SA) and 94 Moderate Asthma (MA) children was performed. The MIMIC approach was applied to investigate the effect of SA and non-controlled asthma (NC) on QoL and behavioural disorders. Logistic regression was used to estimate probabilities of having an “abnormal/borderline” status with variation in QoL. The MIMIC model showed that the magnitude of the effect of SA and NC was larger on QoL (β = −0.37 and β = −0.30, respectively) than on behavioural problems (β = 0.27). With regards to the probability of having a borderline status, in MA a QoL of 1 returned a probability of 0.81, whereas in SA a QoL of 1 returned a probability of 0.89. In conclusion, SA children are highly affected by impaired QoL and behavioural problems. The MIMIC model allowed us to obtain a comprehensive assessment of QoL and behavioural problems with asthma severity and control.

## Introduction

Asthma is one of the most common chronic illnesses of childhood. In clinical practice, physicians are generally focused on diagnosis and treating the respiratory aspects of the disease; however, there are other factors, including psychological facets, which contribute to the disease course, including quality of life (QoL). Children with persistent asthma are at higher risk than healthy children of worse QoL and behavioural problems, which are both related to asthma severity and symptom control^1–3^. In a previous study, we showed worse QoL in children and adolescents with severe asthma (SA) than in those with non-severe persistent asthma^4^. A reduced QoL has also been found in children with non-controlled asthma (NC) compared to children with well-controlled (WC) asthma^5,6^. Measuring QoL in children with asthma can add important information to achieve a fuller picture of children with unrecognized behavioural problems.

Behavioural problems in children are generally described as “internalizing”, which includes “anxiety, depressive, and somatic symptoms” or “externalizing”, which includes “oppositional, hyperactive conduct”^7^. Both internalizing and externalizing problems can be experienced by healthy children with abnormal and “borderline” personality disorder, the latter referring to a mental illness marked by an ongoing pattern of varying moods, self-image, and behaviour^8^. Previous studies on asthma subjects suggests that the magnitude of reported difficulties in behavioural adaptation increased with asthma severity and that behavioural problems may be expressed primarily in the internalizing domain, whereas externalizing problems are affected but at a lesser extent^9,10^. As a whole, these findings suggest that children with asthma, particularly those with severe asthma, should be considered at higher risk for behavioural difficulties that may necessitate psychosocial evaluation.

The relationship between QoL and behavioural problems in paediatric asthma has so far yielded inconsistent results. Tibosch *et al*. reported negative significant correlation between QoL and behavioural problems in children with different levels of asthma severity^11^, whereas Annett *et al*. did not find a correlation between QoL and behavioural problems in mild-moderate asthmatics^9^. To date, based on our knowledge, no study has investigated the relationship between QoL and behavioural problems at various asthma control levels. Therefore, the relationship between QoL and behavioural problems in asthmatics deserves closer investigation, simultaneously taking into account asthma severity and control.

Inter-relationships among subjective and objective asthma outcomes make it difficult to assess the overall effect of covariates or their inter-relationship using standard tests for comparing proportions or logistic regression models. The Structural Equation Model (SEM) is the most suitable statistical tool to address a complex relationship, such as interrelated dependence of QoL, behavioural disorders, and asthma severity and control in a single analysis^12,13^. In particular, the confirmatory factor analysis (CFA) is a type of SEM that studies the relationships between observed measures and latent variables, considering adjustments for correlated measurement error^14^ and the explanatory variables, that is the multiple indicators multiple causes model, or MIMIC. MIMIC models assist in understanding the correlations between various outcomes, latent variables, and potential covariates. Additionally, these models allow detection of direct associations between covariates and outcomes. MIMIC models have been implemented in other medical research areas^15,16^, but they have not yet been applied to research in the field of QoL in children.

The aim of our study was to assess the relationship between QoL and behavioural problems (latent variables) and asthma severity and control (observed measures) through the application of a MIMIC model. The secondary aim was to predict children with a “borderline” behavioural status at varying QoL scores, given the asthma severity level. This was pursued by a tool able to identify a “borderline” status starting from the PAQLQ score.

## Results

### Characteristics of the study sample

Table 1 reports the characteristics of children also studied according to asthma severity. In comparison with MA, SA patients had an earlier asthma onset, more frequent not-controlled, and a large number of oral systemic steroid courses and emergency visits due to asthma exacerbation during the last 12 months. In addition, a lower QoL total score and higher SDQ scores were found in SA patients in comparison with MA patients. In particular, higher scores in both internalizing domain and their subscales (emotional and peer problems) were observed in SA. No patient had an abnormal status as evaluated according to the total SDQ score, while a borderline status was found in a large, though not significant, percentage of SA patients (41.30 *vs*. 26.60, p = 0.117) (Table 2).

**Table 1:** Characteristics of the study sample (no.=141).

#	All	SA	MA	p-value
	141	47	94	
Age, years	10.00 (2.70)	9.80 (2.70)	10.00 (2.55)	0.854
Gender: Male n (%)	87 (61.70)	31 (65.96)	56 (59.57)	0.581
BMI, kg/m^2^	18.11 (5.25)	19.11 (5.76)	17.87 (4.95)	0.238
Family affluence scale, n (%)				0.550
Low	16 (11.59)	6 (13.04)	10 (10.87)	
Medium	62 (44.93)	23 (50.00)	39 (42.39)	
High	60 (43.48)	17 (36.96)	43 (46.74)	
Asthma onset, years	3.00 (4.00)	1.00 (4.00)	3.00 (3.75)	**<0.001**
GINA, n (%)				**<0.001**
WC	40 (28.37)	15 (31.91)	60 (63.83)	
PC	26 (18.44)	8 (17.02)	18 (19.15)	
NC	75 (53.19)	24 (51.06)	16 (17.02)	
*Atopy*	127 (90.07)	45 (95.74)	82 (87.23)	0.196
*Lifetime Comorbidity*				
Rhino-conjunctivitis	81 (57.45)	30 (63.83)	51 (54.26)	0.366
Eczema	19 (13.48)	10 (21.28)	9 (9.57)	0.098
Therapy in the last six months^¥^				**<0.001**
ICS	44 (31.21)	4 (8.51)	40 (42.55)	
ICS-LABA	56 (39.72)	25 (53.19)	31 (32.98)	
ICS + LTRA	14 (9.93)	2 (4.26)	12 (12.77)	
ICS-LABA + LTRA	27 (19.15)	16 (34.04)	11 (11.70)	
OMALIZUMAB	12 (8.51)	12 (25.53)	0 (0.00)	**<0.001**
Number of systemic steroid courses*	0.00 (2.00)	2.00 (3.50)	0.00 (1.00)	**<0.001**
mean (SD)	1.60 (2.92)	3.02 (3.82)	0.88 (2.02)	
Number of emergency visits*	0.00 (1.00)	0.00 (1.00)	0.00 (0.00)	**0.017**
mean (SD)	0.60 (1.47)	0.96 (1.84)	0.41 (1.21)	
Number of hospitalizations*	0.00 (0.00)	0.00 (0.00)	0.00 (0.00)	0.115
mean (SD)	0.23 (0.75)	0.34 (0.92)	0.17 (0.65)	

Data are presented as median and (IQR) or no. (%); ICS: Inhaled Corticosteroids; LABA: Long-Acting Beta Agonists; LTRA: Leukotriene Receptor Antagonists; WC: Well Controlled; PC: Partially Controlled; NC: Non-Controlled; ^¥^BDP equivalent; *Last 12 months.

**Table 2:** PAQLQ, SDQ and MARS scores in the studied subjects.

#	All	SA	MA	p-value
	141	47	94	
**PAQLQ**				
Symptoms Domain	6.60 (1.30)	5.70 (2.15)	6.80 (0.60)	**<0.001**
Activity Limitation Domain	6.80 (1.20)	6.00 (2.60)	7.00 (0.60)	**<0.001**
Emotional Function Domain	6.88 (1.00)	6.00 (2.44)	7.00 (0.25)	**<0.001**
Total score	6.70 (1.08)	5.98 (2.30)	6.85 (0.46)	**<0.001**
**SDQ-P**				
Externalising Domain	4.00 (6.00)	5.00 (6.00)	4.00 (6.00)	0.136
Conduct problems subscale	2.00 (2.00)	2.00 (2.00)	2.00 (2.00)	0.767
Hyperactivity subscale	3.00 (4.00)	4.00 (2.75)	2.00 (4.00)	0.100
Internalising Domain	4.00 (5.00)	6.00 (5.50)	3.00 (4.75)	**0.001**
Emotional problems subscale	3.00 (3.00)	3.00 (3.75)	2.00 (4.00)	**0.010**
Peer problems subscale	1.00 (3.00)	2.00 (3.00)	1.00 (2.00)	**0.005**
Total difficulties score	9.00 (10.25)	12.00 (11.75)	8.00 (10.00)	**0.008**
**Classes of Total difficulties score, n (%)**				
Borderline status	44 (31.43)	19 (41.30)	25 (26.60)	0.117
Normal	96 (68.57)	27 (58.70)	69 (73.40)	
**MARS-9**	44.00 (4.00)	44.00 (4.00)	43.00 (4.00)	0.251

Data are presented as median (IQR) or n (%), p-values come from Kruskal Wallis test.

### Correlations between quality of life and behavioural problems

Figure S1 reports the Spearman correlations of domains and subscales of PAQLQ and SDQ in SA. All domains and subscales were negatively correlated at a significant level, which implies that when the PAQLQ score increases, the SDQ score decreases. Conversely, no significant correlations of domains and subscales of PAQLQ to SDQ in MA were reported.

### Confirmatory factor analysis

Table S1 describes the CFA results, including goodness-of-fit indices, factor loadings for QoL (0.91 for symptom domain, 0.92 for activity limitation domain, 0.96 for emotional function domain) and for behavioural disorders (0.59 for externalization domain, 0.94 for internalization domain) and factor correlation between the two latent factors (QoL, behavioural problems) (r = −0.43, p < 0.001). The model had good fitting in terms of CFI = 1.00 and TLI = 1.00, and acceptable fitting in terms of RMSEA = 0.00 (0.00–010). Overall, based on factor loadings and goodness-of-fit indices, the considered domains for QoL (symptoms domain, activity limitation domain, emotional function domain) and behavioural problems (externalization domain, internalization domain) well explained the latent factors, i.e. QoL and behavioural problems.

### Multiple indicators multiple causes (MIMIC) model

**Figure 1:**
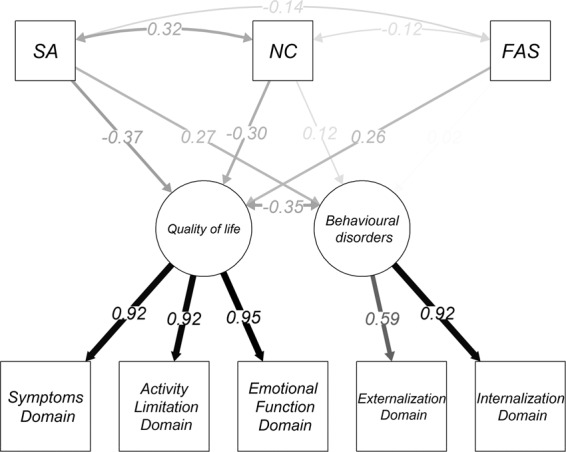
Multiple Indicator Multiple Causes (MIMIC) model showing the impact of background variables on the two factors.

When the selected covariates (Severe Asthma, Not Controlled and Family Affluence Scale) were added, the model fit slightly declined, though staying within acceptable ranges, and the factor loadings remained strong and significant (Table S1), suggesting adequate goodness-of-fit. The standardized effects of the covariates on the latent factors are reported in Table 3 and in Fig. 1. The impact of asthma severity was higher on QoL (β = −0.37) than on behavioural problems (β = 0.27). NC and FAS did not have any significant effect on behavioural problems; conversely, NC had a significant negative effect on QoL (β = −0.30). By contrast, a higher FAS had a significant positive impact on the QoL (β = 0.26). Furthermore, SA and NC were significantly correlated (r = 0.32).

**Table 3:** Effect of asthma severity, asthma control and Family Affluence Scale (FAS) on quality of life and behavioural disorder.

	β	SE	p-value
**Quality of life**			
Asthma severity: SA	−0.37	0.21	**<0.001**
Asthma control: NC	−0.30	0.20	**<0.001**
FAS	0.26	0.07	**<0.001**
**Behavioural disorders**			
Asthma severity: SA	0.27	0.22	**0.006**
Asthma control: NC	0.12	0.20	0.207
FAS	0.02	0.07	0.857

### Sensitivity analysis

A MIMIC model including gender (Female *vs*. Male), comorbidity (No *vs*. Yes) and disease duration (years) was also computed. However, such variables did not show any significant effect on either QoL or behavioural problems. Effect estimates and their statistical significance did not substantially change after including the aforementioned covariates. Indeed, the impact of asthma severity was higher on QoL (β = −0.34) than on behavioural problems (β = 0.21). NC and FAS did not have a significant effect on behavioural problems, whereas NC had a significant negative effect on QoL (β = −0.29). Higher FAS had a significant positive impact on QoL (β = 0.23). The full model reported acceptable fitting in terms of CFI = 0.95 and TLI = 0.93, and unacceptable fitting in terms of RMSEA = 0.08 (0.05–0.12). Therefore, since model fitting did not improve and effect magnitude did not change, reduced model with the selected covariates (SA, NC and FAS) was retained as the best one.

### Estimated probabilities

**Figure 2:**
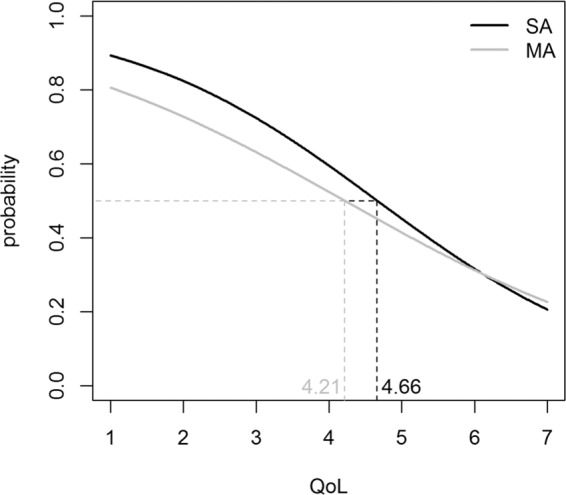
Estimated probabilities from logistic regression model of having a “borderline” SDQ total score (between 14 and 16) with variation in QoL in SA (black curve) and in MA (grey curve).

The probabilities of having a “borderline” SDQ total score (between 14 and 16) with variation in QoL given asthma severity are reported in Fig. 2. The probabilities decreased when the QoL score increased; in MA, a score of QoL between 1 and 4.21 returned a probability between 0.81 and 0.50 of having “borderline” SDQ; in SA a score of QoL between 1 and 4.66 returned a probability between 0.89 to 0.50 of having “borderline” SDQ.

## Discussion

This study provides new information on the relationship between QoL and behavioural problems in patients with moderate and severe asthma. Quality of life impairment and internalizing problems (and, to some extent, externalizing ones) are significantly more prevalent in SA children compared to MA ones. Negative correlations of all PAQLQ domains with SDQ subscales were only found in SA children. Overall, these findings suggest that a psychological assessment should be included in the clinical follow-up of asthmatic children, especially in those with a higher level of severity.

Our study adds more evidence to the previous research, confirming the relationship between QoL and behavioural problems. An added value was the use of a MIMIC model, which showed QoL to be equally affected by asthma severity and uncontrolled status; by contrast, a slightly lower effect of the above factors on behavioural problems was detected. Moreover, the MIMIC model provides evidence that the Familial Affluence Scale influenced QoL, but no effect was recorded on behavioural problems.

Similarly to our previous findings on patients with different levels of asthma severity^4^, in the current study all domains of QoL were significantly more impaired in SA compared to MA. This result is not in line with the finding by Furtado *et al*. that in a smaller sample of asthmatic children reported no significant differences between intermittent/mild and moderate/severe asthmatics, with regard to emotional function and activity limitation domains^17^. This discrepancy is possibly due to the fact that children with moderate and severe asthma were included in the same group, leading to a possible underestimation of the two domains.

To date, few studies have investigated the relationship between behavioural problems and asthma severity, showing that severe asthmatics are at a higher risk of emotional problems^18^, especially internalizing ones^19,20^. Current research suggests that the relationship between asthma and behaviour problems are quite complicated due to the multifactorial determinants of both asthma and behaviour. Difficulties in managing potential asthma stressors (i. e. the need to adhere to daily treatment) mainly related to severe asthma, may represent challenges in behavioural adjustment, giving grounds for a bidirectional disease-behaviour relationship.

Notably, our finding of a significant correlation between QoL and the SDQ domains only in SA patients were limited to the emotional subscale, which refers to unhappiness, nervousness, fear and worries. Similarly, in adolescents with mild-to-moderate asthma, Tibosch *et al*., found significant correlations between QoL and SDQ self-reported for emotional problems and hyperactivity, but no correlations between QoL and conduct problems^11^. Childhood is a specific period in which children show a dynamic behavioural spectrum; especially in those chronically ill, maladaptive behavioural traits may influence multiple outcomes, including health. Therefore, our results suggest integrating clinical and psychological evaluation of the children in routine asthma care.

The application of the MIMIC model allowed us to show that QoL is affected by asthma severity and control, behavioural problems are affected by asthma severity and FAS influences only QoL. These findings suggest that asthma severity is the primary determinant for impaired QoL and behavioural problems. From the clinical point of view, although asthma control is the main goal of asthma management, asthma severity has a major role in influencing both QoL and behavioural problems. Since this approach has never been applied to such data, our findings can only partially be compared with previous studies which investigated this relationship marginally. In fact, with regard to asthma severity, some authors have reported significant effects on QoL^21,22^, and an increased risk of behavioural problems when asthma severity increased^1,19,23^. About asthma control, some studies show that uncontrolled status negatively affected only QoL^24,25^, whereas no study has investigated the effect on SDQ. Some authors show that socioeconomic status of parents was significantly associated with QoL scores in asthmatic children^26,27^, but no study has ever investigated the effect of socioeconomic status on behavioural problems in asthmatic children.

Borderline personality disorder is a mental illness marked by an ongoing pattern of varying moods, self-image, and behaviour. People with borderline personality disorder may experience anger, depression, anxiety and problems in relationships^8^. A “borderline” status has possible transitions over time both to “normal” and “abnormal” status, and this change depends on several factors such as gender, age and socio-economic status of parents^28^. Assessment of behavioural problems requires a considerable amount of time and skilled specialists, representing a limitation for using the SDQ-P questionnaire in clinical practice. Moreover, identification of borderline status could help physicians to plan tailored psychological monitoring, within asthma management, in borderline patients. We have developed a model to predict the borderline status probability, starting from the QoL score given asthma severity, in order to provide clinicians with a tool able to suggest when it is advisable to administer SDQ-P.

Some limitations of the present study should be acknowledged. Firstly, the cross-sectional design; future study should adopt a longitudinal design to address whether the relationship between QoL and behavioural problems persists over time. A second limitation is related to the small sample size, although this is similar to that of previous European studies^4,29^.

The main strength of the study is the choice of a statistical approach such as the MIMIC model. Indeed, the MIMIC model extends the confirmatory factor analysis and path models to allow covariates for simultaneous evaluation of correlations between the latent constructs and among the indicators and the explanatory variables. The resulting latent constructs serve as outcome measures in determining associations with either QoL or behavioural problems, allowing a comprehensive evaluation of the considered phenomenon. In addition, the study used psychometric standardized and validated questionnaires.

## Conclusions

In conclusion, children with severe asthma can be affected by impaired QoL and behavioural problems. The study for the first time allows comprehensive assessment of the complex interrelationship existing between QoL and behavioural problems, suggesting that a psychological assessment should be included in the clinical follow-up of children with severe asthma. Finally, although the estimated probabilities need to be validated in a larger population, they are potentially useful in clinical practice in cases in which the available information only concerns QoL. Future research should focus on validation of the prediction model in other populations and also test the association between QoL and behavioural problems in a longitudinal way.

## Material and Methods

### Study design and participants

This multicentre cross-sectional study was promoted and supported by the Italian Paediatric Respiratory Society (IPRS). The study was approved by the ethics committee of the coordinating center (Anna Meyer Pediatric University Hospital, Florence; approval number: 165/2015); subsequently, it was approved by each local Institutional Ethics Committee.

Informed written consent was obtained from the parent/legal guardian of each child prior to the study inclusion. All children agreed to take part in the study, which was conducted in accordance with Good Clinical Practice and the Declaration of Helsinki.

Participants (aged 6–11 years) included 47 children with SA and 94 with Moderate Asthma (MA), consecutively recruited in the outpatient pulmonary clinics of pediatric departments participating in a national registry of severe asthma. According to ERS/ATS definition, SA is “asthma which requires treatment with high dose inhaled corticosteroids (ICS) doses plus a second controller (and/or systemic corticosteroids) to prevent it from becoming ‘uncontrolled’ or which remains ‘uncontrolled’ despite this therapy”^30^. MA was classified according to Global Initiative for Asthma (GINA) guidelines (www. ginasthma.org) (for a more detailed description of the study population see Supplementary Material).

### Measurements

#### Asthma control

The definition of asthma control status (WC; partly controlled, PC; NC) was performed according to GINA (www. ginasthma.org).

#### Pediatric asthma quality of life

All children were assessed by well-trained physicians with regard to their self-perception of QoL in the “last week”, using the Italian version of the Paediatric Asthma Quality of Life Questionnaire (PAQLQ)^31^. PAQLQ investigates three domains: symptoms, limitations of activity and emotional function: the total score is calculated as the mean of the three domains mentioned. For each domain, the score ranges from 1 to 7, 1 being the worst QoL score and 7 the best QoL score.

#### Behavioural problems

Behavioural problems in the last six months were assessed through the Italian version of the Strengths and Difficulties Questionnaire-Parents (SDQ-P)^32^ which investigates emotional problems, conduct problems, hyper-activity-inattention, peer problems, and prosocial behaviour. SDQ-P includes a total score calculated using the first four subscales, ranging from 0 (best score) to 40 (worst score) points. The total scores were compared to the normative scale making it possible to define the cut-off for normal (0–13 points), borderline (14–16 points) or abnormal results (17–40 points)^33^.

#### Assessment of adherence

The Medication Adherence Report Scale (MARS) was assessed using a 9-item questionnaire^34^ filled by parents. Scores for each item were summed to give a total score ranging from 9 to 45, where higher scores indicate higher levels of reported adherence to the treatment plan.

#### Family affluence scale

The Family Affluence Scale (FAS) was computed using four items: (Item 1) Car: does your family own a car, van or truck? (No = 0; One = 1; Two or more = 2); (Item 2) Own bedroom: do you have your own bedroom for yourself? (No = 0; Yes = 1); (Item 3) Holidays: during the past 12 months, how many times did you travel abroad on holiday with your family? (Never = 0; Once = 1; Two or more = 2); and (Item 4) Computers: how many computers does your family own? (None = 0; One = 1; Two or more = 2). The FAS score was calculated by summing all answers and categorized as low = 0–3, medium = 4–5, high = 6–7^35^.

#### Sample size calculation and statistical analysis

The sample size for the two groups was determined on the basis of a previous study on asthmatic children^4^, where the PAQLQ score in SA was 5.9 (2.3–7.0) and 6.6 (3.7–7.0) in children with non-severe persistent asthma. Detecting the same difference with a 90% statistical power and a 5% significance level would have required a minimum sample size of 34 children for SA and 68 for MA.

Quantitative variables were compared between SA and MA using the Kruskal Wallis test. Differences of categorical variables were analysed using the Chi-squared test.

Confirmatory Factor Analysis was carried out to test a model with two factors, given that limitation of activities, symptoms and emotional problems should weigh on the QoL factor, whereas the internalization and externalization should weigh on the behavioural disorders factor.

Model fitting was evaluated using the comparative fit index (CFI)^36^, Tucker Lewis index (TLI)^37^, and root mean square error of approximation (RMSEA)^38^. For CFI and TLI, values above 0.90 were considered as acceptable fit, and above 0.95 as good fits. An RMSEA below 0.10 was considered as an acceptable fit, and below 0.05 as a good fit.

After assessing the goodness-of-fit of the model, we estimated a MIMIC model including asthma severity (MA as reference), asthma control (categorized as Well Controlled (reference) *vs*. Not Controlled: obtained from Partially Controlled plus Not Controlled) and FAS as covariates.

To check the robustness of our MIMIC model, a sensitivity analysis was performed. The analysis consisted in the simultaneous estimation of the three following regression models, using a/the SEM approach:

(i) Two regression models consisting of two correlated latent factors (QoL and Behavioural disorder):
  $${\rm{QoL}} \sim {\rm{symptoms}}+{\rm{activity}}\,{\rm{limitation}}+{\rm{emotional}}\,{\rm{function}}$$
  $${\rm{Behavioural}}\,{\rm{disorder}} \sim {\rm{externalization}}+{\rm{internalization}}$$
(ii) Regression of explanatory variables on the latent factors:

$${\rm{QoL}}+{\rm{Behavioural}}\,{\rm{disorder}} \sim {\rm{SA}}+{\rm{NC}}+{\rm{FAS}}$$

The significance of the effects was tested using a Z-test.

A p-value < 0.05 was considered significant. Analyses were performed using the R package (3.3.2) statistical analysis software; the MIMIC model was computed using the *lavaan* R package^39^.

## Supplementary information

Supplementary information.
