# Supplementary material for: Relationship between quality of life and behavioural disorders in children with persistent asthma: a Multiple Indicators Multiple Causes (MIMIC) model
Source: Sci Rep. 2020 Apr 24;10:6957. doi: 10.1038/s41598-020-62264-9 (PMC7181655; doi:10.1038/s41598-020-62264-9)
Supplement: Supplementary file 1 — Supplementary information. [file 41598_2020_62264_MOESM1_ESM.doc]

**Supplementary material**

**Relationship between quality of life and behavioural disorders in children with persistent asthma: a Multiple Indicators Multiple Causes (MIMIC) model.**

**Laura Montalbano^#^, PhD^1^ Giuliana Ferrante^#^, PhD^2^, Silvia Montella, MD^3^, Giovanna Cilluffo, PhD^1^*,* Antonio Di Marco, MD^4^, Sara Bozzetto, MD^5^, Emanuela di Palmo, MD^6^ , Amelia Licari, MD^7^, Lucia Leonardi, MD^8^, Valeria Caldarelli, MD^9^, Michele Ghezzi, MD^10^,**  **Stefania La Grutta^¥^, MD, PhD^1^ Franca Rusconi^¥^, MD**^11^ on behalf of the Italian Pediatric Severe Asthma Network (IPSAN) Program of Italian Paediatric Respiratory Society (IPRS)

**^#^ These authors contributed equally to this work**

**^¥^ This author jointly supervised this work**

^1^ National Research Council of Italy, Institute for Research and Biomedical Innovation, IRIB, Via Ugo La Malfa 153, 90146, Palermo, Italy;

^2^ Department of Health Promotion, Mother and Child Care, Internal Medicine and Medical Specialities, University of Palermo

^3^ Department of Translational Medical Sciences, Federico II University, Via Sergio Pansini 5, 80131, Naples, Italy.

^4^ Pediatric Pulmonology and Sleep & Long Term Ventilation Unit, Academic Department Pediatric Hospital “Bambino Gesù”, Piazza S. Onofrio 4, 00165, Rome, Italy

^5^ Division of Emergency Medicine, Department of Women's and Children's Health, University of Padova, Padova, Italy.

^6^ Pediatric Unit, Department of Medical and Surgical Sciences, University of Bologna, 40138 Bologna, Italy

^7^ Pediatric Clinic, Fondazione IRCCS Policlinico San Matteo, University of Pavia, piazzale Golgi 19, 27100, Pavia, Italy;

^8^ Department of Paediatrics, "Sapienza" University of Rome, Rome, Italy

^9^ Department of Mother and Child, 42123 Azienda USL-IRCCS di Reggio Emilia, Italy

^10^Department of Pediatrics, Ospedale dei Bambini, University of Milan, Italy
^11^ Unit of Epidemiology, 'Anna Meyer' Children's University Hospital, Viale Pieraccini 24, 50139 Florence, Italy

**Corresponding author**

Giovanna Cilluffo

Institute for Research and Biomedical Innovation, IRIB

National Research Council (CNR)

Via Ugo La Malfa, 153

90146 Palermo, Italy

Tel: +039 091 6809680

E mail: [giovanna.cilluffo@irib.cnr.it](mailto:giovanna.cilluffo@irib.cnr.it) (GC)

**Methods**

Children aged 6-11 years had been assessed for eligibility at each pediatric outpatient clinic by trained pediatric pulmonologists between January 2017 and December 2017. Each center recruited 2 MA for each SA. The inclusion criteria for SA children were as follows: (1) age 6-11 years; (2) clinical history of bronchial obstruction with bronchodilator reversibility ≥12%; (3) treatment with age-specific high dose of inhaled corticosteroids (ICS) at least for the last 6 months, plus at least another controller (Long-Acting Beta agonists, LABA; Leukotriene receptor antagonists, LTRA) or Omalizumab or systemic corticosteroids (CS) ≥6 months; (4) evaluation and management at a specialized center at least for 4 months during the last 12 months.

The inclusion criteria for MA children were as follows: (1) age 6-11 years; (2) clinical history of bronchial obstruction with bronchodilator reversibility ≥12%; (3) treatment with age-specific medium dose ICS at least for the last 3 months, or low dose of ICS with controller (LABA/LTRA); (4) evaluation and management at a specialized center at least for 4 months during the last 12 months.

For both SA and MPA, the exclusion criteria were as follows: (1) medical diagnosis of cystic fibrosis or ciliary dyskinesia; (2) pulmonary diseases other than asthma; (3) pulmonary anomalies.

Parents of all the enrolled subjects were interviewed by means of a modified version of the SIDRIA questionnaire [1], including enquiries regarding comorbidities, such as lifetime eczema and rhino-conjunctivitis. At the study entry, body mass index (BMI) was calculated as weight (kg)/height (m) squared. BMI was standardized according to WHO <https://www.who.int/growthref/who2007_bmi_for_age/en/>. Additional enquiries concerning lifetime sensitization assessed by skin prick test, number of systemic steroid course, number of emergency visit and number of hospitalizations during the last 12 months.

***Data retrieval and monitoring***

For data collection, a centralized Web-based system (<http://asmadifficile.cineca.it/>). was developed by the CINECA Inter University Consortium (Bologna, Italy) based on secure AXMR® technology. Registered centers accessed the database directly online using a personal identification and password. The system automatically performed eligibility checks and then confirmed or refused the patient’s enrolment. Data were entered by centers on online electronic forms and stored at the quality and security procedure-certified CINECA Data Center. Each center was authorized to have access only to the data of their enrolled patients. The web-reporting system was always available to analyze data, with information daily updated. Data managers (S.M. and F.R.) were properly trained to use the web-data management system.

Figure S1 Correlation plots in SA and in MA. The color of correlations in square (darker square) indicates higher correlation in absolute value), and the first number is the estimated correlation coefficients, the second on between bracket the p-value. The square color represents the direction of correlation (red = positive correlation, blue = negative correlation).


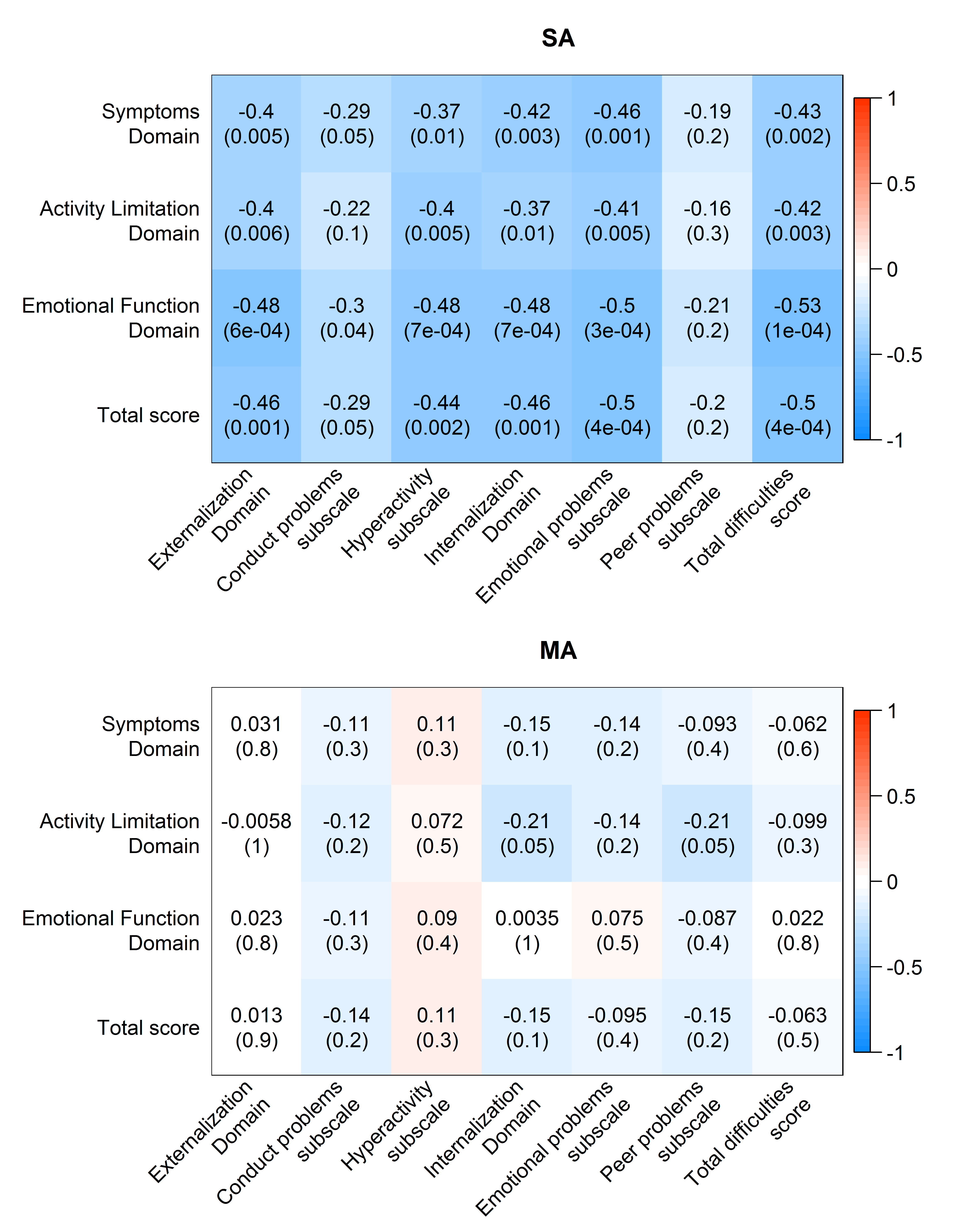


Table S1 Factor loadings, factor correlation and fit indices from CFA and MIMIC

|  | CFA | MIMIC |
| --- | --- | --- |
| **Quality of life** |  |  |
| Symptoms | 0.91 | 0.92 |
| Activity limitation | 0.92 | 0.92 |
| Emotional function | 0.96 | 0.95 |
|  |  |  |
| **Behavioural disorders** |  |  |
| Externalising | 0.59 | 0.59 |
| Internalising | 0.94 | 0.92 |
|  |  |  |
| *Factor correlation* | -0.43 | -0.35 |
|  |  |  |
| *Model fit* |  |  |
| RMSEA (90% CI) | 0.00 (0.00 - 0.10) | 0.04 (0.00-0.10) |
| CFI | 1.00 | 0.99 |
| TLI | 1.00 | 0.99 |

CFA: Confirmatory factor analysis; MIMIC: Multiple Indicators Multiple Causes; RMSEA: Root Mean Square Error of Approximation; CFI: Comparative Fit Index; TLI: Tucker Lewis Index.
